# Normal values of aortic dimensions, distensibility and pulse wave velocity in children and young adults

**DOI:** 10.1186/1532-429X-14-S1-O55

**Published:** 2012-02-01

**Authors:** Inga Voges, Michael Jerosch-Herold, Jürgen Hedderich, Eileen Pardun, Christopher Hart, Dominik Daniel Gabbert, Hans-Heiner Kramer, Carsten Rickers

**Affiliations:** 1 Department of Congenital Heart Disease and Paediatric Cardiology, University Hospital Schleswig-Holstein, Kiel, Germany; 2 Department of Radiology, Brigham & Women’s Hospital & Harvard Medical School, Boston, MA, USA; 3 Department for Medical Informatics and Statistics, University Hospital of Schleswig-Holstein, Kiel, Germany

## Background

Aortic enlargement and impaired bioelasticity is a focus of interest in several cardiac and non-cardiac diseases as it can lead to severe cardiovascular complications. Assessment of aortic anatomy and bioelasticity, namely aortic distensibility and pulse wave velocity (PWV), by cardiovascular magnetic resonance imaging (CMR) is accurate and reproducible and can help to identify anatomical and functional abnormalities of the aorta. However, normal CMR values for healthy children and young adults are lacking.

## Methods

Seventy-one heart-healthy subjects (mean age 16.4 ± 7.6 y, range 2.3 - 28.3 y) were examined using a 3.0-T CMR scanner. Aortic cross-sectional areas and aortic distensibility were measured at four positions of the thoracic aorta. PWV was assessed from aortic phase-contrast flow measurements in a segment which includes the aorta between the ascending aorta and the proximal descending aorta. Reference percentile curves for aortic cross-sectional areas, aortic distensibility and PWV were computed with the Lambda-Mu-Sigma method by Cole.

## Results

Aortic areas, aortic distensibility and PWV (aortic cross-sectional areas: r= 0.8 to 0.9, p<0.001; aortic distensibility r= -0.43 to -0.62, p<0.001; PWV: r= 0.25 to 0.32, p= 0.047 to 0.009) correlated with height, weight, body surface area, and age. There were no significant sex differences. Percentile curves for aortic cross-sectional areas, aortic distensibility and PWV according to age are presented (Figure: Example of reference percentiles for PWV in the aortic arch).

**Figure 1:**
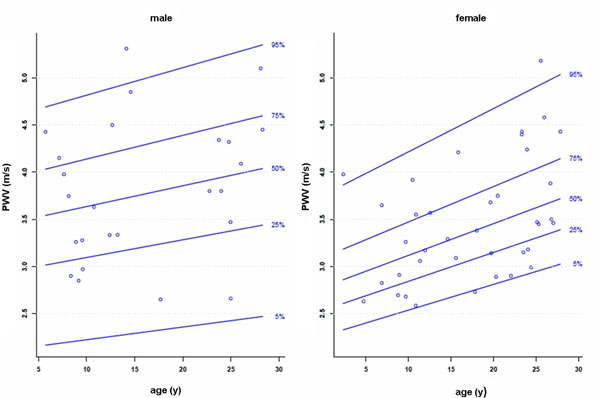


## Conclusions

This study provides percentile curves for cross-sectional areas, distensibility and PWV of the thoracic aorta in children and young adolescents between their 3rd and 29th year of life. These data may serve as a reference for the early detection of pathological changes of the aorta in various cardiovascular diseases.

## Funding

None.

